# Correlates of psychological distress among workers in the mining industry in remote Australia: Evidence from a multi-site cross-sectional survey

**DOI:** 10.1371/journal.pone.0209377

**Published:** 2018-12-20

**Authors:** Carole James, Ross Tynan, Della Roach, Lucy Leigh, Christopher Oldmeadow, Mijanur Rahman, Brian Kelly

**Affiliations:** 1 Centre for Resources Health and Safety, School of Medicine and Public Health, University of Newcastle, Newcastle, Australia; 2 School of Health Sciences, University of Newcastle, Newcastle, Australia; 3 Everymind, Newcastle, Australia; 4 Priority Research Centre for Health Behaviour, School of Medicine and Public Health, University of Newcastle, Newcastle, Australia; 5 Clinical Research Design and Statistics, Hunter Medical Research Institute, Newcastle, Australia; 6 Priority Research Centre for Generational Health and Ageing, School of Medicine and Public Health, University of Newcastle, Newcastle, Australia; 7 Department of Statistics, Comilla University, Comilla, Bangladesh; 8 School of Medicine and Public Health, University of Newcastle, Newcastle, Australia; Australian National University, AUSTRALIA

## Abstract

The purpose of this study was to assess the prevalence of psychological distress in employees in the metalliferous mining industry in Australia, and to examine associated demographic, health, and workplace characteristics. A cross sectional survey was conducted among 1,799 participants from four metalliferous mines. Psychological distress was measured by the Kessler Psychological Distress Scale (K10), alongside other measures of personal demographics, health history, health behaviour, and workplace characteristics. Univariate and multivariate statistical methods were used to examine associations between psychological distress and personal and workplace characteristics. Levels of moderate to very high psychological distress were significantly higher in this sample (44.4%) compared to the general population (27.2%). Moderate to very high psychological distress was significantly associated with younger age; individual health factors (a prior history of depression, anxiety, or drug/alcohol problems); health behaviours (using illicit drugs in the last month); and a range of workplace factors (concern about losing their job; lower satisfaction with work; working shifts of over 12 hours duration; working in mining for financial reasons and social factors (poorer social networks). The identification of a number of social, personal and workplace factors associated with high psychological distress present useful targets to inform the development of tailored workplace interventions to reduce distress in metalliferous mine employees.

## Introduction

It is estimated that mental disorders (including mood, anxiety and substance use disorders), affect up to 1 in 3 people world-wide across their lifetime [1], with the majority of cases occurring in adults of working age [2]. The gap in life expectancy between people with a mental illness and the general population is between 12 and 16 years, with 80% of this attributable to comorbid chronic diseases, many of which are preventable [3]. Not only is this a humanitarian issue, but this also presents a problem for the workplace as most adults spend a third or more of their waking hours at work. The economic and productivity impacts of untreated mental illness and mental ill-health can be substantial for industry with higher absenteeism, presenteeism (less than optimal productivity while at work due to mental health problems), and higher injury rates in those with mental health problems frequently reported [4–8].

In 2010, the direct and indirect estimated cost of mental illness globally was $2.5 trillion, which is projected to more than double to $6 trillion by 2030 [9]. In 2013–2014, the estimated cost of mental illness to both the public and private sectors as well as individuals in Australia was $974 million each year [10]. The annual economic cost owing to lost productivity was even greater, at $11.8 billion [10], highlighting the need to address mental ill-health in society and within industry. The workplace is an ideal setting to implement measures to address mental ill health due to the prevalence of modifiable psychosocial risk-factors in in the workplace, and their association with mental disorders [11, 12]. Workplace mental health interventions have also been shown to provide an economic return on investment for industry [13–15]. Workplace interventions or awareness initiatives can lower rates of depression and presenteeism [14, 16], and lower absenteeism [17]. Understanding the demographic, health, and workplace characteristics associated with mental health problems can improve industry responses and investment. For example, identification of factors contributing to suicide in construction workers has resulted in successful industry investment into a suicide prevention program [18], with resulting economic benefit of $4.60 for every $1 invested [19].

Male-dominated industries such as mining, construction, manufacturing and agriculture are often considered hazardous occupations [20]. The workforce can be highly remunerated, however, the roles are demanding. Typical workplace characteristics often include long shift length, and the work setting is often in rural or remote, geographically isolated locations which can require employees to work away from home, resulting in displacement from family, friends and social networks. The mining industry in particular is a considerable employer in Australia, contributing export earnings of $205 billion in the 2016–2017 financial year, or approximately 6% of Gross Domestic Product [21].

A recent study by the authors reported elevated levels of psychological distress (a state of emotional suffering associated with stressors and demands that are difficult to cope with in daily life [22–24], in a sample of coal mining workers, which was almost 13% higher than a gender and age weighted sample of employed Australians [25]. The study of coal miners also found that those with fewer social connections, those with a previous diagnosis of depression or anxiety, and those who reported hazardous or risky drinking behaviour were at increased risk of high psychological distress. Workplace characteristics identified as impacting upon the mental health of coal miners included concern over losing one’s job, and working for financial reasons. An economic analysis estimated that the annual cost of lost productivity due to psychological distress for the Australian coal mining industry was $153.8 million [26].

There is limited research into how mental health problems differ across different industry settings [27, 28], and no research specifically into the situation within the metalliferous mining sector. Metalliferous mining in Australia is dominated by Fly-in Fly-out (FIFO) workers (whereby workers remain on-site in between shifts, not returning home for weeks at a time) in rural and remote locations where increased distance from health services are observed, and the increased financial and time costs associated with distance have been identified as a barrier to accessing treatment [29]. Stigma regarding help-seeking for mental health issues is also an identified issue in both rural and remote locations [29, 30] and FIFO work sites [31]. Previous research has highlighted the significance of social support and networks in mental health, particularly within rural settings [29, 32]. This is relevant with respect to FIFO workers as research has identified that 40% report feeling lonely or socially isolated and 60% report that the FIFO lifestyle interferes with home and family life [33]. Other reports also identify social isolation as a significant experience of FIFO workers [34, 35]. Substance misuse along with fatigue have also been identified as factors that may be associated with mental health problems in FIFO workers [31, 33].

A psychologically healthy workplace is an organisation where the psychological health of employees is valued and support is provided for those with psychological health problems. Such an organisation is identified as having a strong psychosocial climate [36]. Using components of a workplace psychosocial climate model guided by the Psychosocial Safety Climate theory that advocates the psychosocial safety climate can be predictive of work conditions, worker health and worker engagement [36], the aim of the present study was to assess the prevalence of psychological distress in metalliferous mine workers and explore associated demographic, health, and workplace characteristics. Of particular interest was the exploration of the role of social isolation and remoteness on distress in this population of workers in remote regions, through the impact on social networks.

## Methods

This study was approved by the University of Newcastle Human Research Ethics Committee (approval number: H2013-0135).

### Sample and recruitment

Metalliferous mines across Australia were approached through the Minerals Council of Australia (the peak national industry body) and selected using non-probability quota sampling to ensure a representative cross-section of metalliferous mines across states.

#### Mine site recruitment

The general managers of each mine site were contacted by the research team to gain consent for individual mines to participate. The research team then worked with the Health and Safety managers at each site to determine the logistical arrangements for data collection.

#### Participant recruitment

Promotional material for the study was distributed to each site prior to data collection to raise awareness of the project, then all workers (including contractors and subcontractors) currently working at participating mines were invited to participate in the study.

### Data collection

The data collection procedures were designed to minimise disruption to workplace productivity and to accommodate the unique and specific logistical considerations of each site. The research team attended the site at one of their routine, daily pre-start meetings to deliver a brief presentation outlining: the purpose of the research; that the research was voluntary; that no identifying information was being collected, and that completion or non- completion of the survey did not impact upon employment. Information statements, and paper based surveys were distributed following the presentation and either completed immediately or returned to a ‘post box’ at the mine site in a sealed envelope. Surveys took between 10–15 minutes to complete, with completion implying informed consent. Data was collected between June 2015 and May 2017.

### Measures

#### Psychological distress

The Kessler Psychological Distress Scale (K10) [23] is a 10-item instrument designed to measure participants’ current level of psychological distress. Responses to questions relating to negative emotional states over the preceding four weeks (e.g. in the last four weeks how often did you feel nervous?) are recorded on a 5-point Likert scale ranging from 1 (none of the time) to 5 (all of the time). Total scores were stratified into low (10–15), moderate (16–21), high (22–29) and very high (30–50) categories.

#### Demographic characteristics

Information on age (categorised), gender (M/F), relationship status (single, married/de facto, not married/de facto, separated, widowed, divorced), dependent children (Yes/No) and highest level of education achieved was collected.

#### Individual health history

Participants reported yes or no for any previous diagnosis of chronic conditions (including any of: heart disease, hypertension, hypercholesterolemia, cancer, diabetes, migraine, obesity, stroke), and/or previous diagnosis of mental disorders (including any of: depression, anxiety, or substance use problems).

#### Current health behaviour

Participants were asked to self-report on their current smoking status, as well as the frequency and type of illicit drug use (marijuana, synthetic cannabis, or other illicit substances). Alcohol-use disorders were measured by the 10-item Alcohol Use Disorders Identification Test (AUDIT), a widely used measure of hazardous and/or harmful drinking developed by the World Health Organization. [37]. Reported scores were stratified into categories indicating level of risky drinking behaviour, with scores <8 indicating No Known Risk, and > 8 indicating Risky or High Risk. Social networks were measured as part of current health behaviours. The Social Network Index [38] was used to measure the strength of social and community ties. This measure produces a score based on: the number and frequency of contact with close friends and family; the presence of a spouse or intimate partner; any religious/social participation; and any community group participation. Scores are categorised into four groups: low (low intimate contacts, fewer than 6 friends and no memberships -1), medium (moderately isolated -2), medium/ high (moderately integrated—3) and high (socially integrated -4) with higher scores correlating with higher social and community ties.

#### Workplace factors

Workplace and employment factors include employment status (full-time or part-time); current role (e.g. manager, machinery operator, administrative worker, technician, labourer); number of years working in the mining industry; whether employees identify as locally employed (where they return home at the end of every shift), FIFO, or drive-in, drive-out (DIDO) (defined as either flying or driving to the mine site and living away from home while at work); reasons for working in mining (satisfaction with work, work for financial reasons, the work roster suits my family, and perception of workplace commitment to mental health); length of most common shift (hours); proportion of days spent at work (see Table 1).

**Table 1:** Description of workplace factors and attitudes measured.

Factor	How it was measured
Concern over losing job	A single item measured on a 5-point scale that asked participants to rate their level of concern about losing their job. Scores ranged from 1: `not at all' to 5: `extremely worried'.
Years In Mining	Single item question that determined length of time working in the industry
Employment Category	A single-item question about the employees’-specific occupational role from a list including: manager; professional; technician or trade worker; machinery operator and driver/labourer; or administration/other.
Employment Status	A single-item question that determined if participants worked full-time or part time.
Employment Type	A single-item question to identify participants employed by the mine (principal employee) or as a contractor.
Work schedule (ref = regular shift)	Asked participants to indicate whether they commonly work on a rotating shift pattern (mixture of day/evening/night shifts) or a regular shift (day shift only, or night shift only).
Most common shift length (ref < = 12 hours)	Number of hours of the participant’s most common shift
Days at Work Proportion	Using the participant's typical roster, the proportion of time at work was a ratio of the number of consecutive days at work and the number of consecutive days off work
Financial Reasons	Aggregate score based on average response to three items scored on a 5-point scale ranging from 1: `strongly disagree' to 5: `strongly agree'. Items include: The pay is the main reason I work in coal; I have financial commitments that mean I have to continue to work in coal mining because of the salary levels; I would prefer to work in another job but can't afford to leave because of my financial commitments. (α = 0.73).
Love Work Roster	Average response to two items scored on a 5-point scale ranging from 1: `strongly disagree' to 5: `strongly agree'. Items include: I work in coal because I love the work; the roster schedule suits my family and me. (α = 0.50).
Perception Of Mine Commitment to MH	Average response to five items scored on a 5-point scale ranging from 1: ‘strongly disagree’ to 5: ‘strongly agree’. Items include: this mine would be flexible in offering work adjustments to someone with a mental health problem; this mine provides education and training to supervisors and managers about mental health; the managers at this mine have a good understanding of mental health issues; the mine provides education to employees about mental health; our workplace policies support the mental health of mine employees (α = 0.87).

### Statistical analysis

Descriptive statistics were used to explore the distribution of the sample across demographic and workplace characteristics. Chi-squared tests were used to assess whether the distribution of psychological distress (as measured by the K10) differed from that of the general employed Australian Population taken from the 2007 National Survey of Mental Health and Wellbeing [22], overall and by gender. Chi-squared tests were used to examine the bivariate association of categorical predictors (including participants’ demographic characteristics, individual health history, current health behaviours and workplace characteristics) with the dichotomous outcome variables (combined low/moderate and high/very high K10 scores). To investigate the factors associated with reporting high/very high psychological distress, four multi-variable logistic regression models were performed with the following predictor variables: demographic; individual health history; current health behaviours (including social network characteristics); and workplace factors. Participants’ responses were excluded from all analyses if they did not answer more than 80% of the K10 questions and participants responses were excluded if there was missing data. All the statistical tests were two-sided and significance level was set at *p* < 0.05, adjusted odds ratios are reported for statistically significant associations. A Bonferroni correction to control for the number of multiple regression models that have been fit (*n* = 4) with a threshold of was applied. In addition the Nagelkerke Pseudo R-Square and Area under the ROC curve were calculated for each of the models. The analyses were conducted using SAS 9.4 [SAS Institute, Cary, NC, USA].

## Results

### Participating mines

Four metalliferous mines across Australia participated in the study, one each from Western Australia, Northern Territory, South Australia and Tasmania.

### Mine employees

A total of 1,799 participants from the four sites (Site 1 = 578; Site 2 = 289; Site 3 = 678; Site 4 = 254) completed the survey. Of these, 27 participants were excluded from the analysis as they did not complete 80% of K10 questions. The average response rate across the four mines was 92.3%.

### Sample characteristics

Sample and workplace characteristics are outlined in Table 2. Participants were more likely to be male, aged 25–34, in a married or de facto relationship, indicate that an apprenticeship or trade was their highest level of education, and be working FIFO on a rotating shift (consisting of both day and night shifts) of length 9–12 hours. Participants were also more likely to work in a machinery operator/driver/labourer role within the mine and have been working in mining for between three and 10 years.

**Table 2:** Distribution of demographics and workplace characteristics of the sample (n = 1799).

Personal Variables	*N*	%	Workplace Variables	*N*	*%*
**Sex**			**Mine**		
Male	1575	89.0	1	578	32.0
Female	194	11.0	2	289	16.0
Missing	30		3	678	38.0
**Age**			4	254	14.0
<24	111	6.2	Missing	0	
25–34	665	37.0	**Mine workers**		
35–44	466	26.0	FIFO	1528	85.4
45–54	373	21.0	DIDO	181	10.1
55+	171	9.6	Residential	80	4.5
Missing	13		Missing	10	
**Relationship Status**			**Work Schedule**		
Not Married or not de facto	416	23.0	A Regular Shift	662	37.0
Married or de facto	1242	70.0	A Rotating shift	1074	60.0
Separated/Divorced/Widow	124	7.0	Other	52	2.9
Missing	17		Missing	11	
**Dependent Children**			**Most common shift length**		
No	874	51.0	8 Hours or less	11	0.6
Yes	850	49.0	9–12 hours	1096	61.0
Missing	75		More than 12 hours	683	38.0
**Education**			Missing	9	
Year 10 or less	444	25.0	**Employment Category**		
Year 12	290	16.0	Manager	137	7.7
Trade/Apprentice	550	31.0	Professional	205	11.0
Cert/Diploma	253	14.0	Technician or Tradesman	504	28.0
University/higher degree	245	14.0	Machinery operator and Driver/Labourer	821	46.0
Missing	17		Other + Admin	119 7	6.7
			Missing	13	
			**Years working in Mining**		
			2 Years Or Less	237	13.0
			3 to 10 Years	909	51.0
			More than 10 Years	645	36.0
			Missing	8	

### Psychological distress

**Fig 1:**
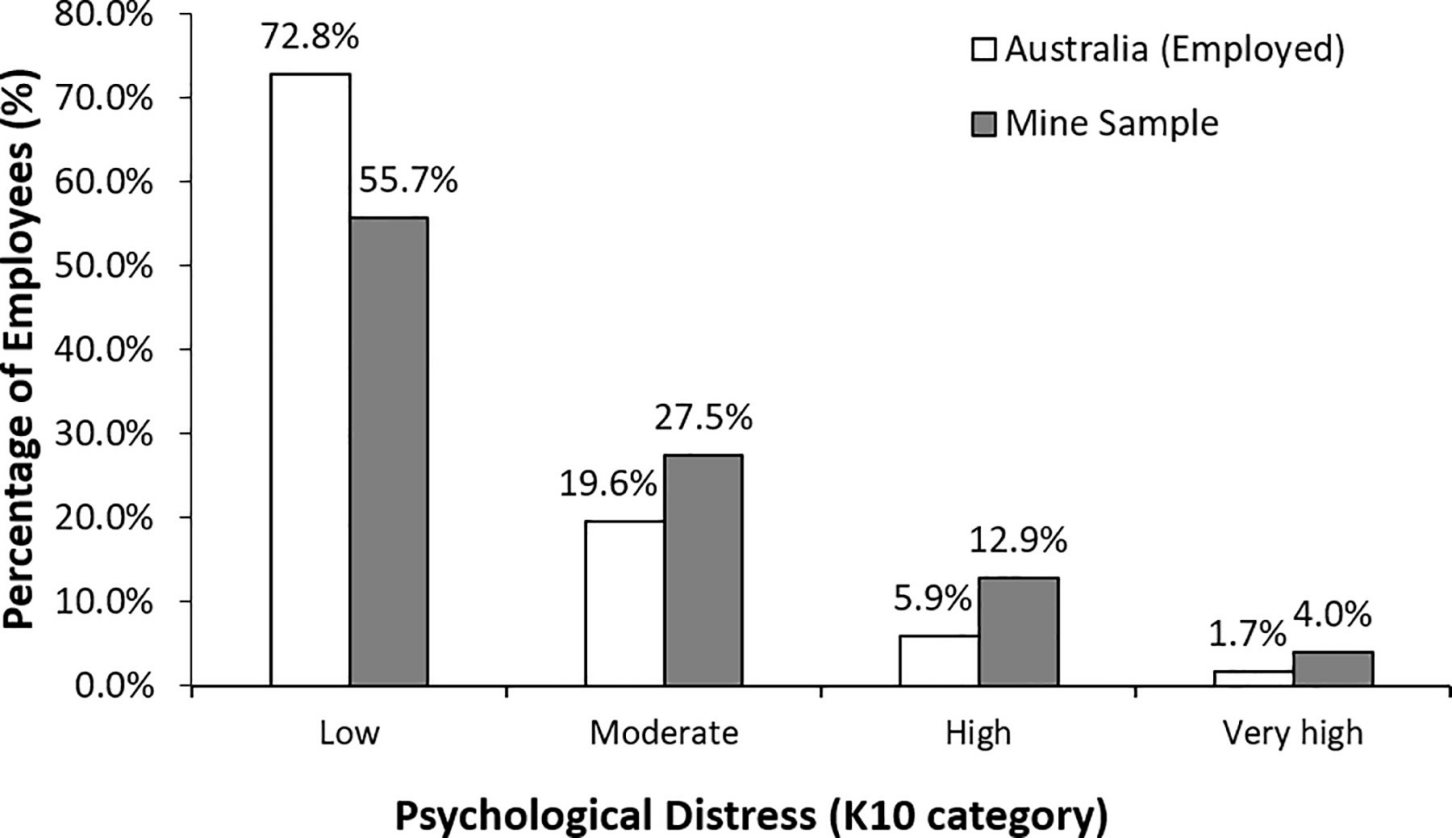
Psychological distress (K10) in metalliferous mines compared with an age and gender weighted sample of employed Australians.

Total K10 scores reported in this sample in each of the strata are shown in Fig 1. A combined 44.4% of the sample reported moderate, high or very high levels of psychological distress. This proportion is significantly greater than the 27.2% reporting the same levels in an gender and age weighted sample of employed Australians (χ^2^(3) = 338.02, *p* <0.001) [22]. Comparing the distributions within gender, the psychological distress levels were higher in the mining sample than the employed Australian sample, with a significant difference for men (χ^2^ (3) = 479.4, p < 0.001), and for women (χ^2^ (2) = 27.3, p < 0.05—note, due to small expected cell counts, the high and very high K10 groups were combined when comparing mining women to employed Australian women).

### Rates of psychological distress by participant and workplace characteristics

Rates of low/moderate and high/very high levels of psychological distress within participants’ demographic, health history, health behaviour and workplace characteristics are shown in Table 3. There was no significant difference in psychological distress between male and female participants. The rates of high/very high psychological distress declined significantly with age, ranging from 23.4% for those aged less than 25 years, to 8.3% for those aged 55 years and over. A significantly higher proportion of those with a Certificate/Diploma reported high/very high psychological distress (25.4%) compared to approximately 15% of employees who have other qualifications including Year 10 or less, Year 12, Trade/Apprentice, and University education or higher. A significantly higher proportion of participants with a history of depression, anxiety or drug or alcohol problems reported high/very high levels of psychological distress compared to those who reported no history (36.4% vs 14.2%; 39.9% vs 13.9%; 34.3% vs 16.3% respectively). Further, the prevalence of high/very high psychological distress was significantly associated with risky/high risk AUDIT scores compared to no risk (20.6% vs 13.5%) and with use of an illicit drug within the last month compared to no drug use (24.0% vs 13.5%). Participants who had any concern over losing their job were significantly more likely to report high/very high levels of psychological distress, as were those who worked a rotating shift (as opposed to a regular shift), those who worked shifts more than 12 hours, and those who had worked in mining for 3 to 10 years.

**Table 3:** Prevalence of psychological distress by characteristics of the sample (n = 1772).

Characteristics	Psychological distress			p-value
	Low/moderate (%)	High/very high (%)	n	
**Gender**				
Male	83.4	16.6	1556	0.71
Female	82.3	17.7	192	
**Age category**				
<25	76.6	23.4	111	
25–34	79.4	20.6	661	<0.001
35–44	81.7	18.3	464	
45–54	89.5	10.5	361	
55 and over	91.7	8.3	168	
**Education**				
Year 10 or less	85.9	14.1	434	
Year 12	83.0	17.0	289	
Trade/Apprenticeship	83.1	17.0	544	0.002
Certificate/Diploma	74.6	25.4	252	
University/higher degree	86.0	14.0	243	
**Chronic conditions**				
No	83.9	16.1	1213	0.133
Yes	81.0	19.0	559	
**Depression**				
No	84.8	14.2	1547	<0.001
Yes	63.6	36.4	225	
**Anxiety**				
No	86.1	14.0	1564	<0.001
Yes	60.1	39.9	208	
**Drug/Alcohol problems**				
No	83.7	16.3	1702	<0.001
Yes	65.7	34.3	70	
**Alcohol use**				
No known risk	86.8	13.2	847	<0.001
Risky or High Risk	79.4	20.6	870	
**Drug use**				
Never/not in the last month	86.5	13.5	1142	<0.001
Yes in the last month	76.0	24.0	584	
**Concern about losing job**				
Not at all	90.2	9.8	559	
Mildly or moderately	83.6	16.5	927	<0.001
Very or extremely	63.2	36.8	201	
**Job Category**				
Manager	84.3	15.7	134	
Professional	85.8	14.2	204	
Technician/Tradesman	83.4	16.6	499	0.53
MO^a^/ Driver/Labourer	81.5	18.5	810	
Other + Admin	85.5	14.5	117	
**Work schedule**				
Regular shift	86.4	13.7	652	0.01
A rotating shift	81.0	19.1	1063	
**Shift length**				
≤ 12 hours	86.7	13.3	1093	<0.001
> 12 hours	77.0	23.0	675	
**Years in mining**				
≤ 2 years	86.5	13.5	230	
3 to 10 Years	80.5	19.5	876	0.02
More than 10 Years	85.0	15.0	661	

^a^ Machine Operator

### Characteristics associated with psychological distress

#### Demographic characteristics

Increasing age was significantly associated with lower odds of reporting high or very high K10 scores compared to reporting low or moderate scores (Aged 45–54 years, OR = 0.41 [95% confidence interval (CI) = 0.22, 0.76]; Aged 55+ years, OR = 0.33 [95% CI = 0.14, 0.72]) (Table 4). Education was also a significant factor with those holding a certificate/diploma two times more likely to report higher K10 scores compared to those who reported completing year 10 or less as their highest level of education (OR = 2.00 [95% CI = 1.32,3.03]).

**Table 4:** Results of multivariable logistic regression models of reporting high and very high psychological distress on demographic characteristics, individual health history and current health behaviours (n = 1,772).

Variables	AOR^a^	95% CI^b^	*p-*value
**Demographic characteristics:** r2 = 0.0301 AUC = 0.627			
Relationship status			
Not married/de facto	ref		
Married and de facto	0.80	0.57, 1.13	0.21
Separated/Divorced/Widow	1.29	0.71, 2.29	0.39
Age Group			
≤24	ref.		
25–34	1.01	0.61, 1.75	0.95
35–44	0.81	0.46, 1.46	0.47
45–54	0.41	0.22, 0.76	0.01
55+	0.33	0.14, 0.72	0.01
Gender			
Female	ref		
Male	1.02	0.65, 1.56	0.90
Dependent children			
No	ref		
Yes	1.14	0.84, 1.57	0.43
Education			
Year 10 or less	ref		
Year 12	1.04	0.67, 1.60	0.85
Cert/Diploma	2.00	1.32, 3.03	0.00
Trade/Apprentice	1.11	0.77, 1.61	0.54
University/higher degree	0.82	0.50, 1.31	0.42
**Individual health history:** r2 = 0.0545 AUC = 0.627			
Chronic physical conditions			
No	ref		
Yes	1.01	0.76, 1.32	0.969
Depression			
No	ref		
Yes	2.05	1.42, 2.95	<0.01
Anxiety			
No	ref		
Yes	2.86	1.97, 4.12	<0.01
Drug/Alcohol Problems			
No	ref		
Yes	2.01	1.15, 3.44	0.01
**Current health behaviours:** r2 = 0.0342 AUC = 0.622			
Alcohol Use			
No known risk	ref		
Risky or High Risk	1.49	1.14, 1.97	<0.01
Smoking status			
Not a daily smoker	ref		
Daily smoker	1.18	0.87, 1.59	0.29
Drug use^c^			
Ever/not in the last month	ref		
Yes in the last month	2.07	1.42, 3.00	<0.01
Social network score			
Low	ref		
Medium	0.62	0.46, 0.82	<0.01
Medium High	0.52	0.34, 0.77	<0.01
High	0.34	0.17, 0.63	<0.01

^a^ Adjusted Odds Ratio

^b^ 95% Confidence Interval

^c^ Due to small numbers of drugs use, the variables relating to cannabis, synthetic drugs, and other illicit substances were combined to ensure adequate model fit

#### Individual health history

A prior history of depression (OR = 2.05 [95% CI = 1.42, 2.95]), anxiety (OR = 2.86 [95% CI = 1.97, 4.12]) or drug or alcohol problems (OR = 2.01 [95% CI = 1.15, 3.44]) were associated with more than double the odds of reporting high/very high K10 scores compared to low/moderate K10 scores (Table 4).

#### Current health behaviours

Combined “Risky” or “High Risk” levels of drinking (compared to drinking at a level associated with no known risk; OR = 1.49 [95% CI = 1.14, 1.97]), and drug use in the last month (OR = 2.07 [95% CI = 1.42, 3.00]) were associated with increased odds of reporting high/very high K10 scores.

Higher social network scores were associated with lower odds of reporting high/very high K10 scores (Medium, OR = 0.62 [95%CI = 0.46, 0.82]; Medium High, OR = 0.52 [95%CI = 0.34, 0.77]; High, OR = 0.34 [95%CI = 0.17,0.63]) (Table 3). This relationship appeared to have a dose-response relationship, in that the higher the social network score, the lower the odds of psychological distress.

#### Workplace factors

Increased concern over losing one’s job (Table 5) (mildly or moderately worried, OR = 1.62 [95% CI = 1.11, 2.40]; very or extremely worried, OR = 3.17 [95% CI = 1.96, 5.16]) was associated with greater odds of psychological distress, as was working for financial reasons (OR = 1.34 [95% CI = 1.12, 1.61]). Having shift lengths longer than 12 hours was also associated with greater odds of high distress (OR = 1.61 [95% CI = 1.17, 2.20]). Conversely, increased satisfaction with work (OR = 0.33 [95% CI = 0.25, 0.43]) and increased perception of the mine’s commitment to mental health (OR = 0.69 [95% CI = 0.55, 0.85]) were associated with decreased odds of high psychological distress. To account for the fact that the proportion of days spent at work needs to be considered in conjunction with whether a participant was FIFO or not, this effect was modelled as an interaction and marginal estimates of the proportion of days at work within each FIFO status are reported. However, the association with reporting greater psychological distress was not significant.

**Table 5:** Results of multivariable logistic regression models of reporting high and very high psychological distress on workplace characteristics (n = 1543).

Variables	AOR^a^		95% CI^b^	*p-*value
**Work place characteristics:** r2 = 0.1662 AUC = 0.803				
Concern about losing job				
Not at all	ref			
Mildly or moderately worried	1.62 ^c^		1.11, 2.40	0.014
Very or Extremely worried	3.17 ^d^		1.96, 5.16	<0.001
Years in mining				
≤ 2 years	ref			
3 to 10 Years	1.38		0.82, 2.39	0.235
More than 10 Years	1.05		0.60, 1.89	0.873
Employment Category				
Manager	ref			
Machinery operator and Driver/labourer	0.65		0.35, 1.24	0.180
Other + Admin	0.84		0.36, 1.91	0.680
Professional	0.78		0.38, 1.64	0.510
Technician or Tradesman	0.84		0.45, 1.60	0.580
Employment status				
Full-time	ref			
Part-time	0.63		0.21, 1.56	0.362
Employment type				
Mine employee	ref			
Contractor/subcontractor	1.29		0.71, 2.25	0.387
Other	1.72		0.08, 12.38	0.638
Work schedule				
Regular shift	ref			
Rotating shift/other	1.34		0.91, 1.97	0.138
Most common shift length				
≤ 12 hours	ref			
>12 hours	1.61 ^c^		1.17, 2.20	0.003
Days at Work Proportion–FIFO^c^	1.11		0.99, 1.26	0.075
Days at Work Proportion–DIDO^d^	0.81		0.03, 23.0	0.897
Days at Work Proportion—Residential	0.5		0.01, 22.2	0.746
*Work attitude factors*				
Satisfaction with work	0.33 ^f^		0.25, 0.43	<0.001
Working for financial reasons	1.34^e^		1.12, 1.61	0.001
Working because I love the work, and the roster suits my family	0.85		0.71, 1.01	0.064
Perception of mine commitment to mental health	0.69 ^e^	0.55, 0.85		0.001

^a^ Adjusted Odds Ratio.

^b^ 95% Confidence Interval.

^c^ Fly-in Fly-out.

^d^Drive-in Drive-Out.

^e^ p < 0.05.

^f^ p < 0.001.

## Discussion

The aim of this study was to investigate the prevalence of psychological distress among the employees within metalliferous mining industry in Australia. The K10 is a widely used measure [39, 40] to screen for clinically significant levels of psychological distress, and likelihood of mental health conditions in the general population. The results of this study show that employees in the metalliferous mining industry report moderate to very high levels of psychological distress at higher levels when compared to a gender and age weighted sample of employed Australians (44.4% compared to 27.2% respectively) [22]. These results are also higher than the 39.1% observed in a comparative sample of employees from the coal mining industry in New South Wales and Queensland [25]. It should be noted that there are differences between types of mining and the mining samples in these studies with both difference in commodity type and in relation to commute type. This metalliferous mining sample had 95.8% of workers employed as FIFO/DIDO whereas the coal sample had only 28.4% in this category with the remaining workers locally employed. A survey of Australian FIFO mine workers in Western Australia also reported average K10 levels higher than employed Australians [41], which supports our findings that Australian mining employees are potentially more susceptible to experiencing higher levels of psychological distress, however is in contrast to our findings where FIFO/DIDO arrangements was not a factor associated with psychological distress specifically. The high rates of psychological distress amongst this population emphasises the importance of addressing mental health problems in the metalliferous mining industry.

Results from this study indicate that factors associated with psychological distress were an interplay of personal and social characteristics and workplace factors. Younger participants, those with a history of depression, anxiety and drug or alcohol problems, those who currently drink at risky or high risk levels, those with fewer social connections and those who reported recent use of illicit drugs were more likely to have higher K10 scores, indicating an increased risk of clinically significant mental health problems. These findings are similar to those from the aforementioned coal mining study, where lower social networks; a past history of depression, anxiety or drug and alcohol problems; and risky alcohol use were all associated with higher psychological distress [25]. Although gender was not an identified characteristic associated with psychological distress, the sample was largely male (89.0%) which is comparable to the male-dominated nature of the mining sector [42]. A systematic review exploring depression in male-dominated industries found eight studies reporting significantly higher levels of depression in male-dominated groups compared to equivalent national data [43], including mining samples. In addition, education level was identified in our study as a characteristic associated with psychological distress, with those participants who identified as having a certificate or diploma education having higher levels of psychological distress than those with a high school education, trade or university degree. This is in contrast to other studies that have identified that mental health problems occur almost equally across all educational and income levels [1, 22]. However, when considering employment category, which is commonly linked with education level, there was no association in levels of psychological stress in our study which concurs with other studies [44, 45]. Of the potentially modifiable factors, stronger social networks were associated with lower levels of distress, paralleling existing findings from rural and remote communities [46] and providing evidence to support a focus on promoting external social connections as an important element in workplace mental health programs for employees working in metalliferous mining. At an internal, workplace level, previous studies have shown that increased social capital in the workplace is associated with a reduction in psychological distress [47], and can positively buffer the effects of job insecurity [48].

Psychosocial safety climate and the associated theory and organisational health framework, incorporates aspects of job design, organisation and management of work and the shared perceptions regarding policy, process and practices with respect to health and safety [49, 50]. These link to the following factors that were included as part of our study. The Participants who reported high psychological distress were significantly more likely to report low satisfaction with their work, greater concern about losing their job, and indicate that they were primarily working in mining for financial reasons. Again, these findings are similar to those from the aforementioned coal mining study, where low satisfaction with work, financial factors, job insecurity and low perceived workplace support for people with mental health problems were all associated with higher levels of psychological distress [25]. With current and anticipated job reductions in the mining sector across Australia, this is not a surprising finding. In Australia and internationally, job insecurity has been commonly associated with adverse health outcomes and in particular, increased levels of mental health problems [12, 25, 51]. For instance, a Canadian study involving mining communities identified increases in mental health problems that correlated with a deteriorating economic climate [52]. Financial stress has also been connected to poor mental and emotional wellbeing, and greater family stress [53].

Employees were also more likely to have higher distress levels if they worked longer shifts and perceived that their employer was not committed to improving mental health in the workplace. Shift length and fatigue are predictors of poor psychological health, especially in mine industry workers [54] and are potential modifiable workplace factors.

In this study, higher levels of psychological distress occurred across all employment categories and there was no significant association between psychological distress and the hours travelled to work or the ratio between days at work and home. This finding contrasts the West Australian Lifeline study of FIFO workers [41], which found higher ratios of work/home were associated with higher levels of psychological distress and poorer coping strategies. The differences in study methodology between the two studies (online vs paper-based survey; and method of study recruitment) and potentially greater diversity between the two samples (for example considerable diversity in work rosters and work sites involved in the Lifeline study) may account for these differences and could be further explored in future studies.

There is limited empirical evidence of how factors outside the workplace might be associated with employees’ mental health [55]. Given that life stressors and significant life events impact on the mental health of the community, it is likely that these community factors also impact on a worker. Similar to this study, a recent Canadian study identified that only 32% of variance was explained by a range of individual and workplace factors on psychological distress [56]. Stansfeld et al. [27] identified that work characteristics alone could not explain why some occupations have higher rates of common mental health problems than others.

The results of this study highlight the importance of personal and social factors on the mental health of mine employees, and points to the need for a broader consideration of mental health in the context of the workplace. It may be that social, workplace and employment characteristics (such as social connection and support, financial strain, shift length and commitment to mental health from employers many of which are components of psychosocial safety climate) provide potentially modifiable factors which could guide interventions that aim to reduce the risk of mental health problems and improve health and safety from a mental health perspective.

### Limitations

This study provides evidence of the extent of mental health problems in the metalliferous mining industry of Australia, however as the study used a cross sectional survey design, causal associations are unable to be determined. Using quota sampling to obtain a non-random sample of participants is also acknowledged as a limitation. Possible bias due to self-report measurement is also acknowledged. In support of the representativeness of the sample there was a strong correlation with industry profile based on the employee age, gender and employment category. It should be acknowledged that respondents voluntarily completed the survey so there may be some volunteer bias within the sample.

The metalliferous mining data was compared with the 2007 data from the Australian National Survey of Mental Health and Wellbeing. It is noted that factors that impact upon the health of the community may have changed since 2007. Since this national community survey, Australia has gone through the global financial crisis, and the mining boom and bust which may have impacted upon individuals and their mental health, both for those employed within the mining industry and those in other industries.

## Conclusion

This study has found that levels of psychological distress in metalliferous mine workers are significantly higher than the average employed Australian worker and support the importance of a focus on mental health within the mining sector. It has also identified a number of social and workplace issues, giving the mining industry an opportunity to target these within appropriate multicomponent workplace interventions that address personal and social factors as well as workplace characteristics. Such interventions should aim to reduce psychological distress with subsequent potential benefits to both individuals and the mining industry as a whole.
